# Unveiling the Identity of Wenwan Walnuts and Phylogenetic Relationships of Asian *Juglans* Species Using Restriction Site-Associated DNA-Sequencing

**DOI:** 10.3389/fpls.2017.01708

**Published:** 2017-10-09

**Authors:** Xian-Yun Mu, Miao Sun, Pei-Fang Yang, Qin-Wen Lin

**Affiliations:** ^1^Laboratory of Systematic Evolution and Biogeography of Woody Plants, College of Nature Conservation, Beijing Forestry University, Beijing, China; ^2^Florida Museum of Natural History, University of Florida, Gainesville, FL, United States; ^3^White Horse Snow Mountain National Nature Reserve Administration, Yunnan, China; ^4^Beijing Botanical Garden, Institute of Botany, The Chinese Academy of Sciences, Beijing, China

**Keywords:** Wenwan walnut, *Juglans hopeiensis*, *Juglans sigillata*, 2b-RAD, phylogeny

## Abstract

*Juglans* species have considerable ecological and economic value worldwide. In China, Wenwan walnuts have been collected by aristocrats and noblemen for more than 2000 years. As a diversity center of Asian *Juglans*, five species are widely distributed in China. The most famous of these is Mahetao (*J. hopeiensis*), which is an uncharacterized species that is mostly cultivated. Wild *J. hopeiensis* individuals are very rare and are endemic to Hebei Province. Because of the minimal variations in previously used molecular markers and the heterogeneity between chloroplast and nuclear genomes, determining the phylogenetic relationships among the *Juglans* species has been challenging, and has hindered subsequent evolutionary inferences. In this study, we collected enough materials for both cultivated and wild Mahetao to construct well-resolved phylogenetic trees for Asian *Juglans* species. We used a high-throughput genome-wide restriction site-associated DNA sequencing method. Consequently, the identity of *J. hopeiensis* has been clearly resolved. Our results indicate that *J. hopeiensis* is a hybrid of *J. regia* and *J. mandshurica*. However, *J. hopeiensis, J. regia* and *J. sigillata* should be considered as a single species from section *Juglans*. Additionally, *J. ailantifolia, J. cathayensis*, and *J. mandshurica* likely represent one species from section *Cardiocaryon* according to morphological and molecular studies. These results are supported by population structure analysis and morphological comparison. We propose that *J. hopeiensis* trees growing in the wild should be conserved because of the economic value of their nuts. These trees may be of particular importance to impoverished communities. Furthermore, they may serve as a valuable genetic resource relevant for enhancing the production of edible walnuts. The 2b-RAD method is a viable option for future phylogenetic studies of *Juglans* species as well as other plant species.

## Introduction

*Juglans* L. is one of the nine extant genera in the family Juglandaceae, and consists of 21 species divided into four sections (Manning, 1978; APG, 2016). In addition to the 17 species distributed in the Americas, there are four species in Asia that are divided into the following two sections: Sect. *Juglans* (e.g., *J. regia*) and Sect. *Cardiocaryon* (e.g., *J. ailantifolia, J. cathayensis*, and *J. mandshurica*). Species in Sect. *Juglans* can be differentiated mainly based on the following traits: number of leaflets (5–11 vs. 7–19), hair on the underside of leaflets (glabrescent vs. glandular pubescent), number of fruits per infructescence (1–3 vs. >5), nut ridges (2 winged vs. 4–8 rough), and number of nut chambers at the base (4 vs. 2) (Manning, 1978). *Juglans regia*, which is also known as English walnut or Persian walnut, is widely cultivated in Europe, from Iran to the Himalayas, and in China because of its considerable economic value as an edible nut. Additionally, its genetic diversity in different growing regions has recently been assessed (Wang et al., 2008; Gunn et al., 2010; Ciarmiello et al., 2011; Pei and Lu, 2011; Dogan et al., 2014; Ebrahimi et al., 2015).

**FIGURE 1:**
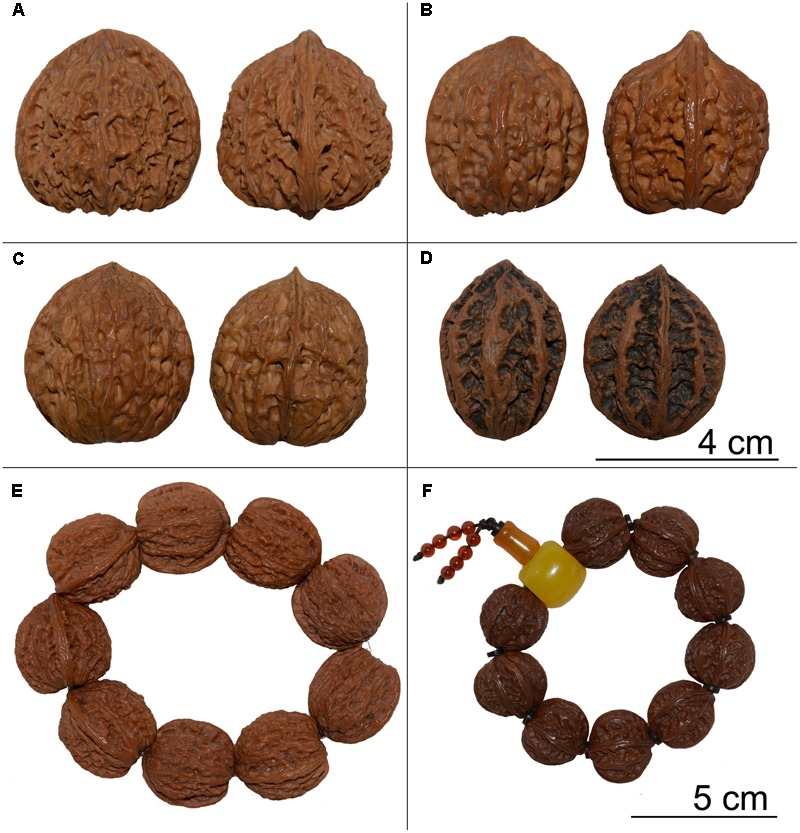
Examples of Wenwan walnut products. **(A–D)**: Front and side views of Wenwan walnuts. **(A,B,E)**: *Juglans hopeiensis*. **(C)**: *Juglans sigillata*. **(D,F)**: *Juglans mandshurica*.

In China, Wenwan walnuts have been considered as playthings among aristocrats and noblemen as early as the Han Dynasty (206 BC–220 AD) (Liu, 2014). In addition to being used for their medicinal qualities, these special walnuts have been collected, offered as gifts, and used to create nut-based sculptures. Consequently, there has been a market for them for more than 2000 years in China. A pair of Wenwan walnuts with the desired size, texture, and color is worth $20,000, which has contributed to the growth of the commercial production of these walnuts (Xi, 2016). There are many Wenwan walnut varieties from different species that are currently marketed, including Hutou, Gongzimao, Guanmao, and Shizitou (**Figure 1**). All Asian *Juglans* nut species have been used as Wenwan walnuts, but the classification and phylogeny of *Juglans* species remains controversial.

Among the *Juglans* species used as Wenwan walnuts, the most famous is Mahetao (*Juglans hopeiensis*), which is an uncharacterized species endemic to Beijing and Hebei Province in China. *Juglans hopeiensis* was first described by Hu (1934) based on two collections. He originally indicated that this species is locally known as Mahetao, and was once quite commonly grown, but it subsequently became less popular. Currently, *J. hopeiensis* is considered to be a rare and conserved plant in Hebei Province in northern China^1^. However, there are still many cultivated walnut trees identified as *J. hopeiensis*, including many varieties, such as Hutou, Gongzimao, Guanmao, and Shizitou. Moreover, there are several opinions regarding the taxonomic position of *J. hopeiensis*. It was initially considered to be a relatively distinct species (Hu, 1934). This belief was supported by microsporogenesis and karyotype analyses (Mu et al., 1990), but not by morphological studies (Manning, 1978; Lu et al., 1999). A second possibility, which was suggested by Prof. Alfred Rehder and mentioned by Hu (1934), is that *J. hopeiensis* is a hybrid between *J. regia* and *J. mandshurica*. This view was widely accepted and supported by some studies based on isozymic and random amplified polymorphic DNA (Cheng and Yang, 1987; Wu et al., 1999). A third possibility proposed by Kuang and Lu (1979) in *Flora Reipublicae Popularis Sinicae* (*FRPS*) is that *J. hopeiensis* is actually a variety of *J. mandshurica*. Recently, an inter-simple sequence repeat-based investigation of the genetic diversity among 138 *Juglans* samples indicated that they represent one entity that is more closely related to *J. mandshurica* than to *J. regia* (Lei, 2010). A recent study that analyzed chloroplast genomic data concluded that *J. hopeiensis* is more closely related to *J. mandshurica* than to *J. cathayensis* (Hu et al., 2017). These studies partly supported the view proposed by Kuang and Lu (1979). Nevertheless, the exact taxonomic position of *J. hopeiensis* and its phylogenetic relationship with other congeneric species remain unclear.

There is yet another Wenwan walnut species, namely *J. sigillata*, which is also called iron walnut. This species was detected by Kuang and Lu (1979), but not by Manning (1978). *Juglans sigillata* is an endemic species distributed in southwest China, and belongs to the Sect. *Juglans* (Lu et al., 1999). It is widely cultivated in the same area as edible walnut. Recent genetic studies indicated that *J. sigillata* is indistinguishable from *J. regia*, suggesting they represent the same species (Aradhya et al., 2007; Wang et al., 2008; Gunn et al., 2010). Therefore, the taxonomic and phylogenetic positions of *J. hopeiensis* and *J. sigillata* will need to be determined in future investigations. Their phylogenetic relationships within the genus are currently unknown. Additional research on these nut species may provide useful information with implications for walnut cultivation and conservation.

Single nucleotide polymorphisms (SNPs) generated during restriction site-associated DNA sequencing (RAD-seq) represent a rich source of high-throughput and genome-wide genetic information. These SNPs have been widely used in diverse biomedical and ecological studies, in part because of their ability to discriminate between individuals in a population. This approach has been recently used to study the evolution and phylogeny of closely related species, with promising results (Rubin et al., 2012; Leache et al., 2015; Zimmer and Wen, 2015; Arbizu et al., 2016; Diaz-Arce et al., 2016; Hou et al., 2016; Razkin et al., 2016). A streamlined and flexible approach for RAD genotyping, called 2b-RAD, is also useful for phylogenetic studies. Compared to other RAD-seq methods, the 2b-RAD protocol is simple and cost-effective (Wang et al., 2012; Seetharam and Stuart, 2013; Puritz et al., 2014). Unlike other RAD-seq methods, the 2b-RAD procedure generates many tags with a uniform length because it involves a site-specific endonuclease type IIB restriction enzyme (i.e., BsaXI) that can cut both strands of double-stranded DNA upstream and downstream of recognized sequences. Additionally, the 2b-RAD method can significantly decrease the complexity of the genome. Furthermore, this approach is not dependent on reference genomes, and it provides an excellent fractional representation of the genome because it produces an abundance of high-quality reads. Thus, 2b-RAD is a powerful option for genetic mapping as well as analyses of quantitative trait loci and adaptations.

Previous studies revealed minimal variations between nuclear and chloroplast markers as well as incongruence between the chloroplast and nuclear genomes in *Juglans* species (Stanford et al., 2000; Aradhya et al., 2007; Stone et al., 2009; Bai et al., 2016). This may prevent researchers from constructing phylogenetic trees for subsequent evolutionary inferences. The objective of this study was to reconstruct the phylogenetic relationships of *Juglans* species based on high-throughput genome-wide RAD-seq data, with a particular focus on Sect. *Juglans* and Sect. *Cardiocaryon*. We also aimed to determine the phylogenetic position of Mahetao (*J. hopeiensis*).

## Materials and Methods

### Taxon Sampling, DNA Extraction, and RAD-seq Data Analysis

A total of 40 *Juglans* samples, including all species in Sect. *Juglans* and Sect. *Cardiocaryon* and two samples of *J. nigra* in Sect. *Rhysocaryon*, were included in this study (**Supplementary Table S1**). Two *Carya illinoinensis* samples were chosen as the outgroup. Leaf material from each sample was preserved in silica gel. Total DNA was extracted using the Plant Genomic DNA kit (Tiangen Biotech Co., Beijing, China). The 2b-RAD libraries were constructed using adaptors (5′-NNN-3′) to cohere the digested products as described by Wang et al. (2012). The sequencing was completed using an Illumina HiSeq X Ten platform. Raw reads were trimmed to remove adaptor sequences, and the 3-bp terminal positions of each read were eliminated. Reads with no restriction sites or ambiguous bases (N), low-quality positions (>20 nucleotide positions with a Phred quality score < 20), or long homopolymer regions (>8%) were discarded. High-quality reads of each sample were aligned using the SOAP2 program (Li et al., 2009). A maximum of two mismatches (–v 2) were allowed for each read, and those mapped onto more than one position in the genomic reference sequence were excluded (–r 0). The match mode was set to “find the best hits” (–M 4). The SNPs were filtered with the RADtyping program (Fu et al., 2013) using the following criteria: (1) Polymorphic loci with more than two alleles were deleted; (2) Segregating markers that could be genotyped in at least 80% of the individuals were kept for analyses; (3) All SNPs with a minor allele frequency (MAF) <0.01 were deleted; and (4) Only one bi-allelic SNP at each locus was retained. The filtered SNPs were subsequently used for the phylogenetic and population structure study.

### Phylogenetic Reconstruction

Phylogenetic analyses were completed using the Maximum Likelihood (ML), Maximum Parsimony (MP), and Bayesian Inference (BI) methods in the RAxML (Stamatakis, 2014), PAUP (Swofford, 2002), and MrBayes 3.2 (Ronquist et al., 2012) programs, respectively. For the MP analyses, heuristic searches were conducted with a random stepwise addition by tree bisection-reconnection (TBR) branch swapping; one tree was held at each step during the stepwise addition, with the MULTrees option turned on. All of the character states were unordered and equally weighted, and gaps were defined as missing data. Bootstrap values from Maximum Parsimony (MP_BS_) were estimated from 1000 replicates in a heuristic search with simple addition using the implemented TBR and MULPARS options. Prior to the ML and BI analyses, a model of sequence evolution for each matrix was determined based on the Akaike information criterion (Posada and Buckley, 2004) using the Modeltest 3.7 program (Posada and Crandall, 1998). The ML analyses were conducted using RAxML v. 8.1.12 with 1000 replicates (ML_BS_) under the GTRCAT model as implemented by HiPerGator 2.0 at the University of Florida. For the BI analyses, the Markov chain Monte Carlo algorithm was applied to each dataset, with three hot chains and one cold chain for 8 × 10^6^ generations in parallel mode. Trees were sampled every 100 generations beginning with a random tree. The run was stopped when the average standard deviation of split frequencies was less than 0.01 in all cases. Bayesian posterior probabilities (BI_PP_) were calculated as the 50% majority-rule consensus of all sampled trees, with the first 20% discarded as burn-in.

### Population Structure Analysis

Because of complicated relationship involved potential gene flow among species in six Asian *Juglans* species, population structure cluster analysis based on STRUCTURE Ver. 2.3.4 software (Pritchard et al., 2000) was performed for samples of Sect. *Juglans* and Sect. *Cardiocaryon* in this study. The SNPs datasets for population structure analysis were selected from the 21,111 SNPs using PLINK v.1.07 software (Purcell et al., 2007). SNPs with MAF < 0.05 were excluded, and then select unlinked loci using the function indep-pairwise (50 10 0.1) were used. The MCMC simulations were used, and three replicate for the number of clusters (*K*) from 1 to 6 were conducted. All the runs were performed using a model of admixture, with a burn-in of 100,000 followed by 200,000 iterations. Structure Harvester web server was used to identify the optimal *K* value (Earl and vonHoldt, 2012). Considering the results from three replicates of the selected *K* value were the same, no further analysis was conducted.

## Results

### RAD-seq Data Matrix

The sequencing of 42 2b-RAD libraries generated 311,049,694 raw reads (mean number of reads = 7,405,945). The total sequencing depth was 33×. After trimming the barcode, cleaning, and filtering out the low-quality reads, we obtained a total of 258,012,168 high-quality reads (i.e., 82.97% of the raw reads). Overall, an average of 39.59% of the high-quality reads for each sample were uniquely mapped (**Supplementary Table S2**). The RAD data have been deposited in the NCBI database (accession numbers SAMN06473106–SAMN06473147). A total of 21,111 SNPs were genotyped and used for our phylogenetic study.

### Reconstruction of Phylogeny

**FIGURE 2:**
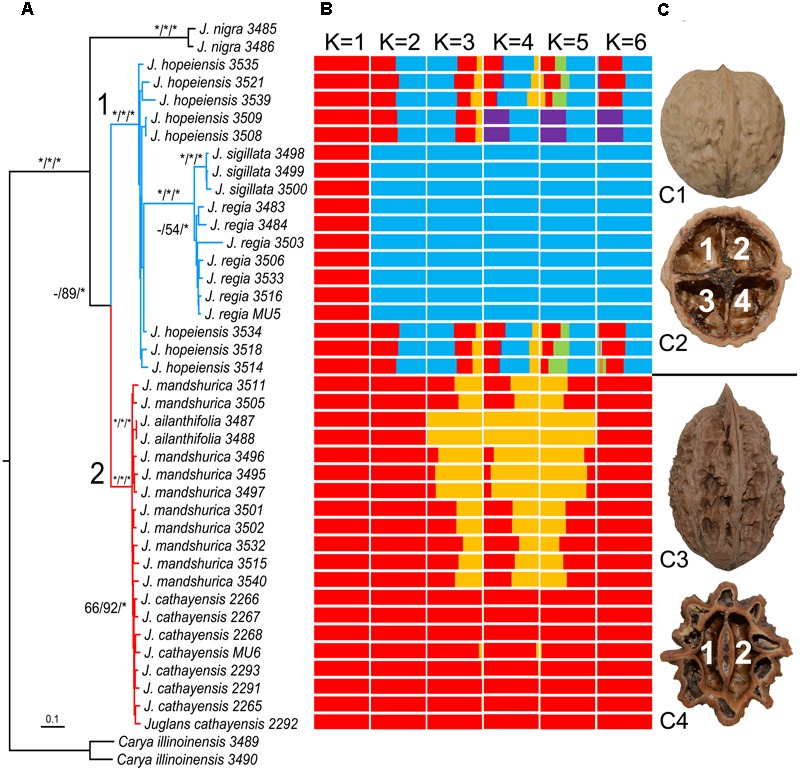
Phylogenetic tree, population structure and nut morphology of Asian *Juglans*. **(A)**: Molecular tree inferred from Maximum Likelihood (ML) analyses using 42-taxa and 21,111 bp RAD-seq data matrix. Bootstrap values ≥ 50% in the Maximum Parsimony (MP) and Maximum Likelihood (ML) analyses and posterior probabilities ≥ 0.90 in the Bayesian Inference (BI) analysis are indicated at the species level on the branches (MP_BS_/ML_BS_/BI_PP_). The asterisk represents the maximum support value (BSs and PP = 100), while the hyphen refers to support values ≤ 50% for MP and ML or PP ≤ 0.9 in BI. The infrageneric classification of *Juglans* species agrees with the results of Manning (1978). **(B)**: STRUCTURE output with *K* = 1–6, and *K* = 2 is the selected value by Structure Harvester. **(C)**: Nuts of *J. regia* in Sect. *Juglans* show two definite ridges on the outer surface (C1) and four internal chambers at the base (C2), and nuts of *J. mandshurica* in Sect. *Cardiocaryon* show eight definite ridges on the outer surface (C3) and two internal chambers at the base (C4).

A matrix with 42 taxa and 21,111 bp RAD-seq data was obtained for our phylogenetic study. The ML analyses conducted in RAxML resulted in a well-resolved *Juglans* topology, which was the same as trees constructed using the MP and BI methods. We herein present the ML tree, with MP_BS_/ML_BS_/BI_PP_ indicated on the branches (**Figure 2A**). Clade support values are presented on the tree at the species level. The North American species were sisters to the Asian species, with full support values (MP_BS_ = 100, ML_BS_ = 100, and BI_PP_ = 1). The Wenwan walnut (*J. hopeiensis*) and species in Sect. *Juglans* (*J. regia* and *J. sigillata*) also formed a fully supported clade. The *J. regia* and *J. sigillata* samples formed a strongly supported clade, and these two monophyletic clades represented sisters (MP_BS_ = 100, ML_BS_ = 100, and BI_PP_ = 1). All species in Sect. *Cardiocaryon* formed a fully supported clade, including two samples of *J. ailantifolia* that nested in the cluster formed by *J. cathayensis* and *J. mandshurica*.

### Clustering

A dataset of 909 SNPs was generated for population clustering study. STRUCTURE analysis strongly favored a two-population model (**Supplementary Figure S1**), which corresponding our phylogenetic result and morphological comparison that only one species are recognized in Sect. *Juglans* and Sect. *Cardiocaryon*, respectively (**Figures 2B,C**). What is interesting is that, the suspected hybrid species, *J. hopeiensis*, is clearly demonstrated to be a hybrid of *J. regia* and *J. mandshurica*.

## Discussion

### Phylogenetic Relationships within Asian *Juglans* Species

In this study, the phylogenetic relationships among Asian *Juglans* species were resolved using SNP data generated using a RAD-seq approach. Our data revealed that North American and East Asian species are divided into two clades. Among the East Asian species, there were two subclades, corresponding to the two sections proposed in a morphological study, namely Sect. *Juglans* (including *J. regia* and *J. sigillata*) and Sect. *Cardiocaryon* (including *J. ailantifolia, J. cathayensis*, and *J. mandshurica*). Furthermore, the previously uncharacterized Mahetao (*J. hopeiensis*) together with *J. regia* and *J. sigillata* were resolved in Sect. *Juglans*, with full support from the MP, ML, and BI analyses, combing population structure cluster analyses and fruit morphological similarity.

We re-evaluated the phylogenetic relationships within Sect. *Juglans*. *Juglans regia* and *J. sigillata* were considered as distinct species in *FRPS* and *Flora of China*, while recent molecular genetic diversity studies indicated differentiating between these two species is difficult (Wang et al., 2008; Gunn et al., 2010). Moreover, these two species were nested in a strongly supported monophyletic clade in phylogenetic studies using chloroplast markers (Aradhya et al., 2007; Hu et al., 2017). However, it should be noted that in the previous phylogenetic studies, *J. regia* and *J. sigillata* were represented by only one sample each. In contrast, we included seven *J. regia* samples collected from different areas and three *J. sigillata* samples. Our results indicate that the samples of each species formed strongly supported clusters. Furthermore, their close phylogenetic relationship is also presented by the STRUCTURE analysis. However, these samples were embedded with eight *J. hopeiensis* samples to form a strongly supported monophyletic clade (**Figure 2A**). The boundary of *J. hopeiensis, J. regia*, and *J. sigillata* is obscure, and the fact they clustered in a monophyletic clade representing Sect. *Juglans* was inconsistent with the results from previous studies. However, a hybrid identity of *J. hopeiensis* is clearly presented by STRUCTURE (**Figure 2B**). The *J. hopeiensis* samples included in this study comprised two wild individuals and six grafted individuals from their main production sites in Beijing (i.e., Mentougou, Changping, Miyun, and Yanqing districts). The divergence in the branch lengths of *J. hopeiensis* and the two edible walnut species in Sect. *Juglans* was expected because of the long history of human selection during the cultivation of two edible walnut species. Additionally, most of the *J. hopeiensis* individuals had been grafted without sexual reproduction.

**FIGURE 3:**
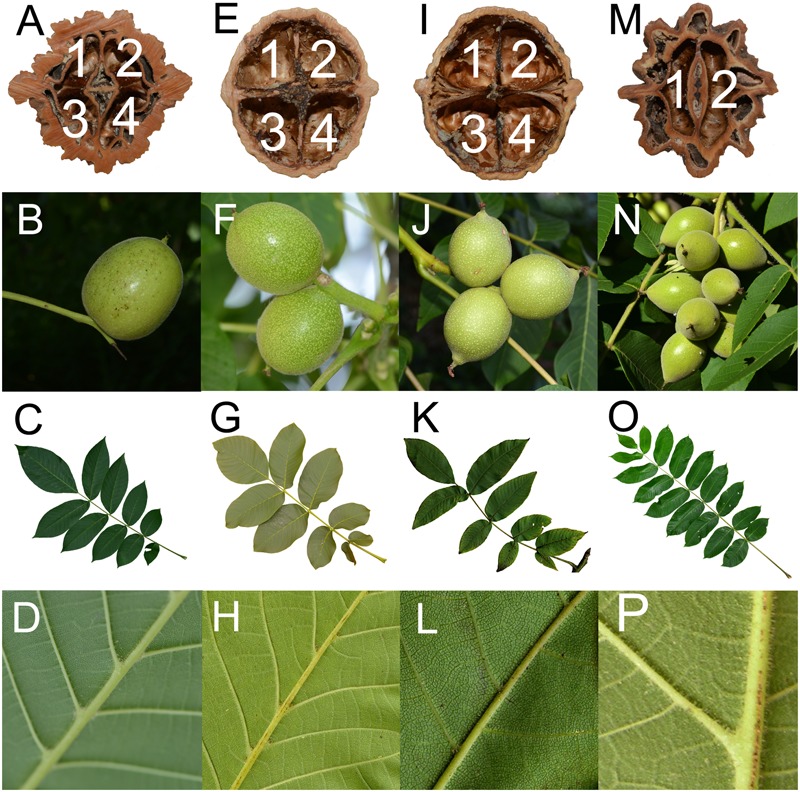
Morphological comparison of *Juglans hopeiensis, J. regia, J. sigillata* and *J. mandshurica*. It is four-chambered nuts, 1–3 fruits on infructescences, 9–11 leaflets, and glabrescent veins on the underside of leaflets for *Juglans hopeiensis, J. regia*, and *J. sigillata* and two-chambered, >5 fruits on infructescences, 9–23 leaflets, and pubescent veins on the underside of leaflets for *J. mandshurica*. **A–D**: *J. hopeiensis*; **E–H**: *J. regia*; **I–L**: *J. sigillata*; **M–P**: *J. mandshurica*.

In addition to the molecular evidence, morphological characteristics also indicated that *J. hopeiensis* is similar to the two edible walnut species, *J. regia* and *J. sigillata*. The results of our detailed morphological comparison of the nuts, infructescences, and leaves are presented in **Figure 3**. Differences in the number of nut chambers and ridges were obvious between Sect. *Juglans* (i.e., four cells and two ridges) and Sect. *Cardiocaryon* (i.e., two cells and four or eight ridges) (Manning, 1978). The *J. regia* fruits were sometimes thick, and contained two air chambers inside the septum, similar to the fruits of *J. mandshurica*. Although covered by a thick shell and lacunate septa, similar to the nuts of Sect. *Cardiocaryon*, the *J. hopeiensis* nuts consisted of roughly two ridges on the outer surface and an incomplete four-celled internal chamber at the base (**Figure 3A**). The species in Sect. *Juglans* produced 1–3 fruits per infructescence, unlike the species in Sect. *Cardiocaryon*, which produced more than five. *Juglans hopeiensis* produced the same number of fruits as the species of Sect. *Juglans* (**Figure 3B**). According to *FRPS*, the Sect. *Juglans* species have 5–11 leaflets, while the Sect. *Cardiocaryon* species have 7–25 leaflets, and *J. hopeiensis* produces 7–15 leaflets. However, based on our field observations, individual *J. hopeiensis* trees at most collection sites had 9–11 leaflets (**Figure 3C**), similar to *J. sigillata* trees. Furthermore, an obvious difference in leaf characteristics was that species in Sect. *Juglans* produced glabrescent leaflets while species in Sect. *Cardiocaryon* produced leaflets with glandular hairs. In contrast, the *J. hopeiensis* leaflets under field conditions were frequently observed to be glabrescent or glabrous on the veins on the underside of leaflets, similar to the species in Sect. *Juglans*. Additionally, the *J. hopeiensis* leaflets lacked the dense and persistent glandular hairs observed on leaflets from species in Sect. *Cardiocaryon* (**Figure 3D**).

Our phylogenetic analyses using RAD-seq data and population cluster analyses using STRUCTURE confirmed that *J. regia* and *J. sigillata* should be treated as a single species as suggested in previous studies (Aradhya et al., 2007; Wang et al., 2008; Gunn et al., 2010). Furthermore, our data appeared to imply that *J. hopeiensis* should also be included in that Sect. *Juglans*. The hybrid identity of *J. hopeiensis* is demonstrated by our deep molecular phylogenetic and STRUCTURE inference, and whole chloroplast genome phylogenetic study (Hu et al., 2017). *Juglans hopeiensis* may represent a wild-type variety of *J. regia* that does not undergo sexual reproduction because of human interference that has preserved potentially important genetic resources for the development of edible walnuts. Unfortunately, there are relatively few old wild-type trees growing under natural conditions. Because their nuts are desired as collectables and have high economic value, *J. hopeiensis* trees may be useful for impoverished communities in remote mountainous regions in China, which is the natural habitat for this tree species. Therefore, protecting the *J. hopeiensis* trees growing in the wild should be a high priority.

**FIGURE 4:**
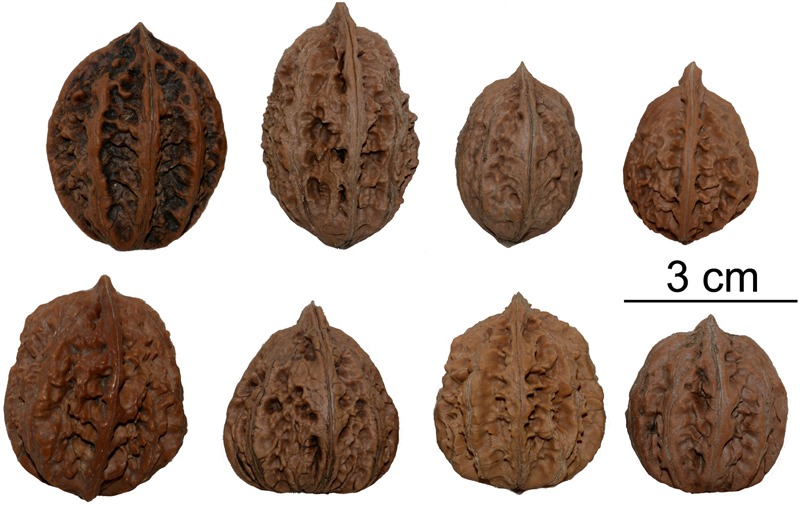
Complex nut morphology in *Juglans* Sect. *Cardiocaryon*, including irregular variations in nut volume, shape, and outer ridges and pits.

We investigated the relationships among the species in Sect. *Cardiocaryon*, and observed that *J. mandshurica*, which is distributed in northern China, and *J. ailantifolia*, which is grown in Japan, may be the same species. This differs from the conclusions of an earlier phylogeographic study (Bai et al., 2016). The possibility that *J. cathayensis* is monophyletic is supported with moderate values (MP_BS_ = 65, ML_BS_ = 92, BI_PP_ = 1), which is consistent with the findings of Bai et al. (2016). The leaf morphological characteristics, irregular and complex variations in fruit morphology (**Figure 4**), and the phylogenetic results described in this study indicate that *J. cathayensis* and *J. mandshurica* are the same species, as suggested in *Flora of China*.

### Utility of RAD-seq in a Phylogenetic Study of *Juglans* Species

Because of the variable fragment lengths generated in commonly used RAD-seq methods, the SNPs data matrix contains a large number of missing data in related phylogenetic studies. Strategies developed to compensate for the missing data may result in different scenarios (Aberer et al., 2013; Hipp et al., 2014; Hou et al., 2016). However, other studies have indicated that the proportion of missing data in RAD-seq data matrices may have minimal effects on the accuracy of phylogenetic inferences (Rubin et al., 2012; Hou et al., 2015). In this study, we applied the 2b-RAD method, which generates fragments with a uniform length. Phylogenetic trees generated from MP, ML, and BI analyses exhibited the same topology, and all were strongly supported. Although incongruence exists between chloroplast genome and nuclear genomes in *Juglans*, which may hinder its molecular phylogenetic inference, our RAD-seq method is a good choice and the result is echoed by population cluster analysis. Therefore, the 2b-RAD approach represents a viable option for future phylogenetic studies of *Juglans* species. This method may also be useful for investigating the evolution of other plant species.

## Author Contributions

Q-WL is the corresponding author, he proposed this study subject, designed the relevant experiments, and also partially funded this study. X-YM is the first author and corresponding author, and his contributions are including sample collection, experiment performing, data analysis, paper writing and also partially funded this study. MS is the second author, and He mainly contributed to data analysis and paper modification. P-FY mainly contributed to collecting some very valuable walnut samples.

## Conflict of Interest Statement

The authors declare that the research was conducted in the absence of any commercial or financial relationships that could be construed as a potential conflict of interest.

## Footnotes

1 http://rep.iplant.cn/protlist/9?key=Juglans%20hopeiensis

## Supplementary Material

FIGURE S1 DeltaK generated by Structure Harvester in population clustering analysis.

TABLE S1 Sample information.

TABLE S2 Information of RAD-seq data.
