# Supplementary figures and images for: Unveiling the Identity of Wenwan Walnuts and Phylogenetic Relationships of Asian Juglans Species Using Restriction Site-Associated DNA-Sequencing
Source: Front Plant Sci. 2017 Oct 9;8:1708. doi: 10.3389/fpls.2017.01708 (PMC5641410; doi:10.3389/fpls.2017.01708)

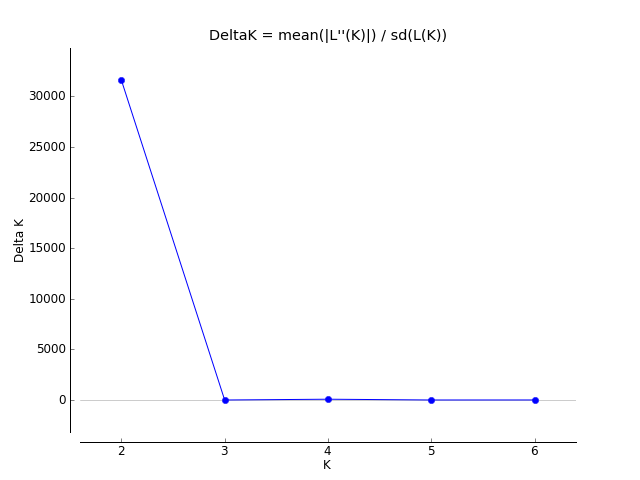

Supplement: FIGURE S1 — DeltaK generated by Structure Harvester in population clustering analysis. [file Image_1.TIF]
